# A whole-blood transcriptomic analysis of shared molecular mechanisms between subclinical atherosclerosis and hypothyroidism in women

**DOI:** 10.3389/fbinf.2026.1932220

**Published:** 2026-10-21

**Authors:** Emiliana María Torres, Diego Alejandro Raffo, Mariana Lorena Tellechea

**Affiliations:** Centro de Investigaciones Endocrinológicas “Dr. César Bergadá”, Consejo Nacional de Investigaciones Científicas y Técnicas - Fundación de Endocrinología Infantil - División de Endocrinología, Hospital de Niños Ricardo Gutiérrez, Buenos Aires, Argentina

**Keywords:** atherosclerosis, hypothyroidism, IFRD1, macrophages, TSHR

## Abstract

**Introduction:**

Hypothyroidism (HT) is clinically associated with an increased risk of cardiovascular disease, yet the shared molecular mechanisms underlying subclinical atherosclerosis (sAS) in hypothyroid patients remain poorly understood. This study aims to identify common transcriptomic signatures and regulatory axes between these two conditions.

**Methods:**

We performed an integrated transcriptomic analysis of peripheral whole-blood datasets retrieved from the Gene Expression Omnibus (GSE176153 and GSE221615). To minimize sex-based biological bias, the analysis was restricted to female samples. Differential expression, functional enrichment, network topology, and transcription factor prediction were performed to identify shared differentially expressed genes (SDEGs) and candidate transcriptional regulators. The classification performance of selected pivotal SDEGs and predicted transcription factors was assessed by receiver operating characteristic (ROC) analysis. Public transcriptomic data from myeloid-specific Tshr-knockout murine macrophages (GSE275079) were additionally examined to explore the myeloid context of TSH–TSHR signaling, followed by *in vitro* RT-qPCR validation in human THP-1 macrophages exposed to TSH and LPS.

**Results:**

We identified 20 female-restricted SDEGs enriched in pathways related to B-cell proliferation and activation. Integration of functional enrichment, functional network analysis, and PPI topology highlighted six pivotal genes: *BLK*, *CD19*, *CD180*, *TNFRSF13C*, *SPIB,* and *FCRLA*. *SPI1* and *MEF2A* were identified as key candidate regulators collectively targeting this gene set. Exploratory ROC analyses showed discriminatory capacity for several pivotal SDEGs and candidate transcription factors across the study cohorts, with some of these findings also observed in an external cohort of advanced atherosclerotic disease. Analysis of Tshr-knockout murine macrophage data revealed enrichment in lymphocyte and mononuclear cell proliferation pathways, alongside downregulation of *Ifrd1*. In human THP-1 macrophages, TSH alone and in combination with LPS were associated with a significant downregulation of *IFRD1* expression.

**Discussion:**

These findings suggest a potential sex-specific immune-endocrine link between thyroid dysfunction and atherosclerotic disease, characterized by a shared transcriptomic axis converging on B-cell-associated pivotal genes and candidate transcriptional regulators. Exploratory myeloid analyses further identified TSH-associated downregulation of *IFRD*1.

## 1 Introduction

Atherosclerosis (AS) is the primary underlying cause of cardiovascular (CV) diseases, which remain the leading cause of mortality worldwide (Kopych et al., 2025). Atherosclerotic CV disease (ASCVD) encompasses a broad spectrum of disease, from subclinical atherosclerotic plaque to acute events with lifelong morbidity or sudden mortality (Ahlers et al., 2025). As a chronic progressive condition, AS begins early in life and remains clinically silent for extended periods before clinical manifestations appear. Consequently, there is growing consensus that halting the global burden of ASCVD requires a new standard of care targeting AS in its subclinical phase, particularly in younger, asymptomatic individuals (Nielsen et al., 2024).

The initiation and progression of AS are driven by a complex interplay between traditional metabolic alterations, lifestyle, genetic predisposition, and chronic vascular inflammation (Gusev and Sarapultsev, 2023) (Pepin and Gupta, 2024). In women, the risk of ASCVD accelerates during the menopause transition due to hormonal, metabolic, and vascular changes. Both menopause and menopause hormone therapy may further modify CV risk, depending on the timing and characteristics of treatment (D’Costa et al., 2025).

During early atherogenesis, vascular endothelial cells undergo activation and structural dysfunction under insults from chronic oxidative stress, disturbed blood flow, and modified low-density lipoproteins (LDL). This endothelial dysfunction triggers a cascade of innate and adaptive immune events, mediated by the upregulation of specific cell adhesion molecules that recruit circulating leukocytes and T lymphocytes to the intima (Rohde et al., 2022) (Kopych et al., 2025). Within the subendothelial space, monocyte-derived macrophages internalize modified lipids to form foam cells, establishing a persistent inflammatory microenvironment.

In 1856, Virchow initially suggested that the immune system might be involved in the pathogenesis of AS (Mayerl et al., 2006; Ma et al., 2021). While research has historically focused on the roles of monocytes, macrophages, and CD4^+^ or CD8^+^ T cells as the predominant drivers of plaque progression, the systemic contribution of other immune components has recently been revised (Luo et al., 2025). Emerging evidence underscores a critical regulatory or pathogenic role for B cells in atherogenesis (Ueland et al., 2026). Although B cells are relatively scarce in atherosclerotic plaques, they are abundant in the perivascular adipose tissue, in addition to the spleen and bone marrow. Besides, the atherosclerotic vessels can also harbor tertiary lymphoid organs, which contain B cells (Pattarabanjird et al., 2021). According to recent research, bone marrow activation in subclinical atherosclerosis (sAS) appears to be associated with CV risk factors, increased haematopoiesis, systemic inflammation, and arterial macrophage infiltration in humans (Devesa et al., 2022). In this regard, bone marrow activation in response to CV risk factors may trigger the release of haematopoietic progenitors and start the inflammatory process that leads to AS initiation and progression (Devesa et al., 2022). On the other hand, it has been proposed that CV disease remodels the vascular bone marrow niche, stimulating hematopoiesis and production of inflammatory leukocytes (Rohde et al., 2022). At a conceptual level, these data indicate feedback loops that perpetuate a cycle of inflammation and CV disease progression.

In patients receiving statin therapy, residual inflammatory risk (assessed via high-sensitivity CRP) is a stronger predictor of future CV events and mortality than residual cholesterol risk (Alomair et al., 2024; Porsch and Binder, 2024; Ahlers et al., 2025). This supports inflammation as an important therapeutic target in ASCVD. Accordingly, the landmark CANTOS trial confirmed that neutralizing IL-1beta with canakinumab significantly reduces recurrent CV disease (Ridker et al., 2017). Driven by these insights, other targeted anti-inflammatory therapies have recently demonstrated substantial clinical potential in treating ASCVD (Rohde et al., 2022; Ahlers et al., 2025; Luo et al., 2025).

Importantly, hypothyroidism (HT), encompassing both overt and subclinical states, is tightly associated with an elevated risk of developing ASCVD. HT, and especially subclinical HT, correlates with various well-characterized metabolic and vascular comorbidities, including impaired lipid homeostasis, diastolic hypertension, altered coagulation profiles, and endothelial dysfunction, which are established contributors to sAS (Torres and Tellechea, 2025a; 2025b) (Yao et al., 2018).

The primary etiology of HT is Autoimmune Thyroiditis (AIT, or Hashimoto’s thyroiditis), a chronic disorder characterized by thyroid lymphocytic infiltration and fibrosis, as well as positivity for thyroid-specific antibodies (Li et al., 2025). Its pathogenesis reflects a complex interplay among genetic, environmental, and immune factors, with intricate interactions among various immune cells and inflammatory mediators within the thyroid gland (Wang et al., 2026). Notably, there is a marked increase in T and B lymphocytes, which exacerbate the condition by secreting a range of cytokines and chemokines that sustain the chronic inflammatory state in the thyroid and induce structural and functional impairments. AIT shows a strong sex bias, with adult females having approximately four times the risk of AIT compared with adult males (Wang et al., 2026), and frequently co-occurs with systemic autoimmune diseases such as rheumatoid arthritis (RA) and systemic lupus erythematosus (SLE) (Ma et al., 2025).

The pro-atherogenic environment associated with HT may reflect the combined effects of reduced thyroid hormone levels (Rand et al., 2025), direct signaling via elevated thyroid-stimulating hormone (TSH), and underlying systemic immune dysregulation. We have previously demonstrated that HT is characterized by an increase in circulating biomarkers of endothelial dysfunction, CRP and proinflammatory cytokines, and malondialdehyde—an end-product of lipid peroxidation (Tellechea, 2021; Torres and Tellechea, 2025a). This evidence may suggest that an inflammatory loop can cause and perpetuate local and systemic inflammation, forming a vicious cycle leading to endothelial dysfunction and CV disease (Torres and Tellechea, 2025a). However, the exact shared molecular mechanisms and transcriptional networks linking sAS and HT, particularly within women, have not yet been fully elucidated.

In view of the potential overlap in immune-related processes between sAS and HT, characterizing their shared pathological features may provide insight into the biological links between these conditions and potentially inform future therapeutic strategies. Therefore, this study aimed to identify shared transcriptomic signatures and candidate regulatory mechanisms linking sAS and HT using peripheral blood RNA sequencing data from public datasets, with a specific focus on female-specific cohorts. By integrating differential expression, functional enrichment, network analysis, and transcription factor prediction, we sought to identify shared molecular players and regulatory axes that may contribute to the pathophysiological overlap between sAS and HT.

## 2 Materials and methods

### 2.1 Data acquisition and preprocessing

Gene expression datasets for HT and sAS were systematically retrieved from the Gene Expression Omnibus (GEO) database (https://www.ncbi.nlm.nih.gov/geo/; Access Date: 2025.10). The search strategy used “Hypothyroidism” and “Subclinical Atherosclerosis” as primary keywords. Inclusion criteria were: (1) *homo sapiens* species; (2) sample size n ≥ 3 per group; (3) availability of raw or normalized expression data by high-throughput sequencing (HTS); (4) samples were taken from peripheral whole blood. Exclusion criteria included: (1) cell line or animal studies; (2) datasets without healthy controls. Based on these criteria, two primary datasets, GSE176153 (de Almeida et al., 2023) and GSE221615 (Sánchez-Cabo et al., 2023) were selected. The HT-GSE176153 dataset comprised four healthy euthyroid controls and four hypothyroid patients (1 male and three females per group). Notably, HT patients were levothyroxine-naïve, and controls maintained TSH levels within the reference range. The sAS-GSE221615 dataset included 391 samples (364 males and 27 females), consisting of 255 patients with sAS and 136 without sAS (Supplementary Figure 1; Table 1). Additionally, GSE202625 was utilized as an external exploratory dataset, featuring consecutive patients undergoing coronary CT angiography, classified into severe stenosis (n = 27) and subjects without AS (n = 25).

**TABLE 1:** Predicted transcription factors (TFs) and their SDEG targets.

TF	NES	Targets	Evidence
*POU5F1*	6.310	*SOX6, SPIB, GPM6A, CD180*	Motif-based
*SPI1*	6.227	*ARHGAP24, NAPSB, GPM6A, CD1C, BLK, OSBPL10, CD180, MYH6, TNFRSF13C, CD19*	Track-based
*MEF2A*	5.933	*BLK, FCRLA, TNFRSF13C, OSBPL10, ARHGAP24, CD180, SPIB, NAPSB*	Track-based
*IRF3*	5.164	*GPM6A, SOX6, CD19*	Track-based
*CEBPE*	5.055	*SOX6, SPIB, ARHGAP24*	Motif-based
*MEF2C*	5.015	*BLK, FCRLA, TNFRSF13C, OSBPL10, ARHGAP24, CD180, SPIB, NAPSB*	Track-based
*PPARG*	5.005	*MYH6, BLK, CD180*	Motif-based

Transcription factor (TF) enrichment analysis was performed using the iRegulon plugin (v1.3) in Cytoscape. Candidate transcription factors were identified using a Normalized Enrichment Score (NES) threshold >5.0, a rank threshold of 5,000, and a maximum false discovery rate (FDR) on motif similarity of 0.001. Regulatory associations were identified based on predicted transcription factor binding motifs (Motif-based) or experimental ChIP-seq-derived tracks (Track-based).

All raw FASTQ files were retrieved from the European Nucleotide Archive (ENA).

### 2.2 Differential gene expression analysis and identification of common DEGs

All datasets were analyzed separately during the Differential Gene Expression analysis. Raw data were processed using R software (version 4.5.2). The genomic sequence from the BSgenome.Hsapiens.UCSC.hg38 package was utilized to build the reference genome index using the Rsubread package, against which the FASTQ reads were subsequently aligned. The aligned BAM files were then processed to count reads overlapping the gene exons. The analysis of Differential Gene Expression was conducted using the DESeq2 package. For sAS-GSE221615, age and sex were included as covariates in the DESeq2 model. The HT-GSE176153 dataset was analyzed according to its original design, given its limited sample size (n = 8). Transcripts below a row-sum threshold of 10 were filtered. The org.Hs.eg.db package was used for gene annotation. With the screening criteria of p-value <0.05 and |log2FC| > 0.26 (equivalent to a 1.2-fold change), DEGs in the two datasets were identified. The purpose of this analysis was to identify DEGs associated with sAS and HT, providing a foundation for subsequent analyses (Yuan et al., 2023; Zhong et al., 2024). The intersection of DEGs was performed to identify shared DEGs (SDEGs). As a sensitivity analysis, the DEG lists were additionally filtered using Benjamini–Hochberg false discovery rate (BH-FDR)-adjusted p-values (padj <0.05), while retaining the |log2FC| > 0.26 threshold, and the intersection of the resulting FDR-significant DEG lists was re-examined (Gorji and Sadkowski, 2026). Because the HT cohort contained only one male per group, a secondary sex-specific analysis was subsequently performed using female samples only (Supplementary Figure 1; Table 1). For sASq-GSE221615, age was included as a covariate.

### 2.3 Functional enrichment analysis

Gene Ontology (GO) enrichment analysis was conducted with the clusterProfiler 4.18.4 package, with enrichGO function (Wu T. et al., 2021). Parameters were set at a p-value <0.05, q-value <0.2, with Benjamini–Hochberg correction for multiple testing. GO categories were restricted to gene sets containing 10–500 genes (minGSSize = 10, maxGSSize = 500). The default background gene set implemented by clusterProfiler was used for enrichment analysis. To ensure statistical robustness, GO enrichment analysis was only performed for gene lists containing at least 10 genes. Results were visualized through dot plots and chord diagrams. The ClueGO v. 2.5.10 plugin in Cytoscape 3.10.4 was used to demonstrate the internal interconnection of GO BP (30.04.2025 update) terms involved in SDEGs with medium network specificity (Term/pathway selection 4, Kappa 05) (Bindea et al., 2009).

### 2.4 Protein-protein interaction (PPI) and hub gene identification

To investigate the molecular mechanisms underlying sAS and HT, a PPI network was constructed. The SDEGs were imported into the STRING app within Cytoscape, applying a high-confidence minimum interaction score of 0.85. Network topology was evaluated using node degree, and genes with the highest connectivity were considered candidate hub genes.

### 2.5 Identification of regulatory factors

Transcription factor (TF) analysis was performed using the iRegulon (v1.3) plugin in Cytoscape. The SDEGs were analyzed to identify potential transcriptional regulators. TF–target gene pairings were selected based on a Normalized Enrichment Score (NES) > 5.

### 2.6 ROC analysis of pivotal genes and transcription factors

The individual classification performance of the identified pivotal genes and selected transcription factors was evaluated using Receiver Operating Characteristic (ROC) curves and Area Under the Curve (AUC) analysis. ROC analyses were performed using the *pROC* package in R, and graphical representations were generated using *ggplot2*. For each ROC curve, the AUC and corresponding 95% confidence interval (CI) were calculated.

### 2.7 Analysis of immune infiltration

Immune cell deconvolution was performed using the CIBERSORTx machine-learning algorithm to estimate the relative proportions of 22 distinct immune cell types (LM22 signature). To generate Transcripts Per Million (TPM) expressions, pseudo-alignment was executed via Kallisto (version 0.51.1), utilizing the prebuilt *Homo sapiens* index provided by the Pachter Lab. The resulting abundance files were imported and aggregated at the gene level using the tximport package, and biomaRt was utilized for comprehensive gene annotation. For the CIBERSORTx run, parameters were set to 1,000 permutations with quantile normalization disabled. To ensure statistical reliability, downstream analyses were restricted to cell types detected in at least 75% of the total samples and samples with a CIBERSORTx deconvolution p-value <0.05 (Wu X.-G. et al., 2021). Relative immune cell fractions were compared between disease and control groups. Spearman’s correlation analysis was performed to evaluate the associations between pivotal SDEGs and shifting immune cell fractions, visualized via the corrplot (version 0.95) package.

### 2.8 Comparative analysis of shared and distinct molecular pathways with autoimmune disorders

To explore if the shared mechanisms between HT and sAS are unique or common to systemic autoimmunity, we analyzed datasets for Rheumatoid Arthritis (RA) and Systemic Lupus Erythematosus (SLE). The expression profiling data were searched in the GEO database (last search January 2026), filtering the results by study type (expression profiling by HTS) and organism (*Homo sapiens*). We restricted the data collection process to studies involving whole blood samples from patients and controls with a minimum sample size of three per group with available raw data. We downloaded the metadata with the GEOquery R package to apply our inclusion criteria and exclude those samples that do not meet them. After applying these criteria, we finally obtained the following datasets: GSE264125, GSE112087, GSE80183, GSE90081, and GSE117769. Analyses were performed using raw count matrices downloaded directly from GEO.

Additionally, to specifically address autoimmune thyroiditis (AIT), the GSE269497 dataset (n = 10) was included. FASTQ files were retrieved using the SRA Toolkit. By intersecting DEGs from sAS and AIT, we sought to decouple the effects of thyroid hormone deficiency/TSH elevation from the underlying autoimmune process. This may allow us to explore whether the pro-atherogenic environment is primarily driven by the metabolic/hormonal state of HT itself rather than the presence of autoimmunity.

The analytical pipelines for these datasets remained consistent with the protocols described above.

### 2.9 Analysis of TSHR gene expression in whole blood

Considering evidence that the *TSHR* gene is expressed in monocytes and macrophages (Zhang et al., 2025), we conducted a retrospective transcriptomic analysis to further investigate its expression profile in human T and B lymphocytes. A systematic search in the GEO database (last accessed January 2026) was performed, restricted to studies employing HTS. We specifically selected datasets derived from primary, non-cultured human peripheral blood and containing purified or sorted leukocyte populations from healthy individuals, with a minimum threshold of three biological replicates per group. Seven datasets were identified (GSE136200, GSE110159, GSE227743, GSE51984, GSE221091, GSE60424, and GSE107011). Data processing was executed using raw count matrices retrieved directly from GEO, following the analytical pipeline described above and including only normal T-cell, B-cell, and monocyte populations from healthy controls.

### 2.10 Transcriptomic analysis of TSH signaling in murine macrophages

To evaluate the effects of TSHR signaling in myeloid cells, the GSE275079 dataset was retrieved from GEO. Raw RNA-seq data from primary bone marrow-derived macrophages (BMDMs)—isolated from male, myeloid-specific *Tshr* knockout (KO) mice and wild-type (WT) littermates—were downloaded from ENA. Differential expression was performed using DESeq2 (KO vs. WT) with thresholds of adjusted p-value <0.05 and |log2FC| > 1. Downregulated DEGs underwent functional enrichment analysis via enrichGO.

### 2.11 Cell culture

The human monocytic leukemia cell line THP-1 (derived from a male infant) was maintained in Roswell Park Memorial Institute medium (RPMI 1640; Thermo Fisher Scientific, Waltham, MA, United States) supplemented with 10% heat-inactivated fetal bovine serum (FBS; Thermo Fisher Scientific), 100 U/mL penicillin, 100 μg/mL streptomycin (Gibco, Grand Island, NY, United States), and 2 mM glutamine (Gibco) at 37 °C and 5% CO2. THP-1 monocytes were differentiated into M0 macrophages by a 72-h incubation in 48-well plates (at a density of 5 × 105 cells/mL) with 50 nM (30.8 ng/mL) phorbol 12-myristate 13-acetate (PMA, #P8139, Sigma-Aldrich, St. Louis, MO, United States) followed by a 48-h incubation in PMA-free medium. Differentiated THP-1 macrophages represent a widely established *in vitro* model for evaluating human macrophage involvement in inflammatory responses (Park et al., 2007). Cells were then stimulated with 1) TSH (#T8931, Sigma-Aldrich) at two different concentrations with or without activation with lipopolysaccharides (LPS) (50 ng/mL, *Escherichia coli* O111:B4, #L4391, Sigma-Aldrich), 2) LPS alone (positive control), or 3) vehicle for 24 h. TSH was used at 1 and 10 mU/mL based on previous *in vitro* studies (Donnini et al., 2003; Balzan et al., 2012; Zhang et al., 2014; Jiang et al., 2018).

### 2.12 Isolation of mRNA and RT-qPCR

Total RNA was isolated using TRI Reagent® (Sigma-Aldrich, St. Louis, MO, United States). Total cellular RNA was used to synthesize cDNA using SuperScript™ III reverse transcriptase (Invitrogen, Carlsbad, CA, United States) according to the manufacturer’s instructions. Quantitative polymerase chain reaction was performed using a FastStart SYBR Green Master mix (Roche Diagnostics, Basel, Switzerland) as follows: 95 °C for 10 min, 45 cycles of 95 °C for 15 s and 60 °C for 1 min on a StepOnePlus™ Real-Time PCR System (Applied Biosystems, Foster City, CA, United States). The primer sequences used were as follows: *IFRD1* forward, 5′-TGCAGTGGTTATAGCGATCCT-3′ and reverse, 5′- CCTTGTCTTCGCACTCTTATCC-3′; *MEF2A* forward, 5′- AGCAGCCCTCAGCTCTCTTG-3′ and reverse, 5′-GGTGAAATCGGTTCGGACTTG-3′; Glyceraldehyde-3-phosphate dehydrogenase (*GAPDH*) forward, 5′-GGGAAGGTGAAGGTCGGAGT-3′ and reverse, 5′- GGGGTCATTGATGGCAACA-3′. Melting curves were performed to corroborate qPCR signal specificity by a single peak at the desired melting temperature (Tm). The efficiency of the amplification reaction for each gene was calculated by running a standard curve of a serially diluted cDNA sample. In each assay, a control reaction without the cDNA was also subjected to PCR amplification. Gene expression changes were analyzed according to the Pfaffl method. Statistical analyses were performed using R software. Normality was evaluated using the Shapiro-Wilk test. Pairwise comparisons between the control (vehicle) and each treatment group were performed using one-way ANOVA followed by Dunnett’s *post hoc* test. A corrected p-value of less than 0.05 was considered statistically significant.

## 3 Results

### 3.1 Identification of shared DEGs (SDEGs)

An initial comparative analysis between the sAS-GSE221615 and HT-GSE176153 datasets identified 4 SDEGs (*RASSF6, ETV7, PXDNL*, and *DAAM2*). Among these, *RASSF6* and *ETV7* were consistently up- and downregulated, respectively, across both conditions. To account for potential sex-based biological bias, as the HT-GSE176153 dataset contained only one male per group, a secondary sex-specific analysis was performed focusing exclusively on female samples. This HTq-GSE176153 subset consisted of six female samples (3 cases and three controls). Correspondingly, we filtered the sASq-GSE221615 subset from the total cohort (N = 391) to include 27 female samples (13 with sAS and 14 controls; Supplementary Figure 1). The intersection of DEGs from these female-specific subsets revealed 20 SDEGs (Figure 1A). This signature included 15 upregulated (*TNFRSF13C, MYH6, SPIB, GPM6A, OSBPL10, BLK, CD19, SAXO2, SOX6, ARHGAP24, FCRLA, CD1C, DLL3, CD180,* and *NAPSB*), two downregulated (*HS3ST3A1* and *SCGB3A1*), and three discordantly regulated (*ZNF354A, MS4A3*, and *IFRD1*) SDEGs. To assess the robustness of the shared signature to a more stringent significance threshold, we repeated the analyses using BH FDR-adjusted p-values (Supplementary Table 2). Under this criterion, no SDEGs were identified. Therefore, the gene signature identified using the nominal p-value threshold should be considered exploratory and hypothesis-generating.

**FIGURE 1:**
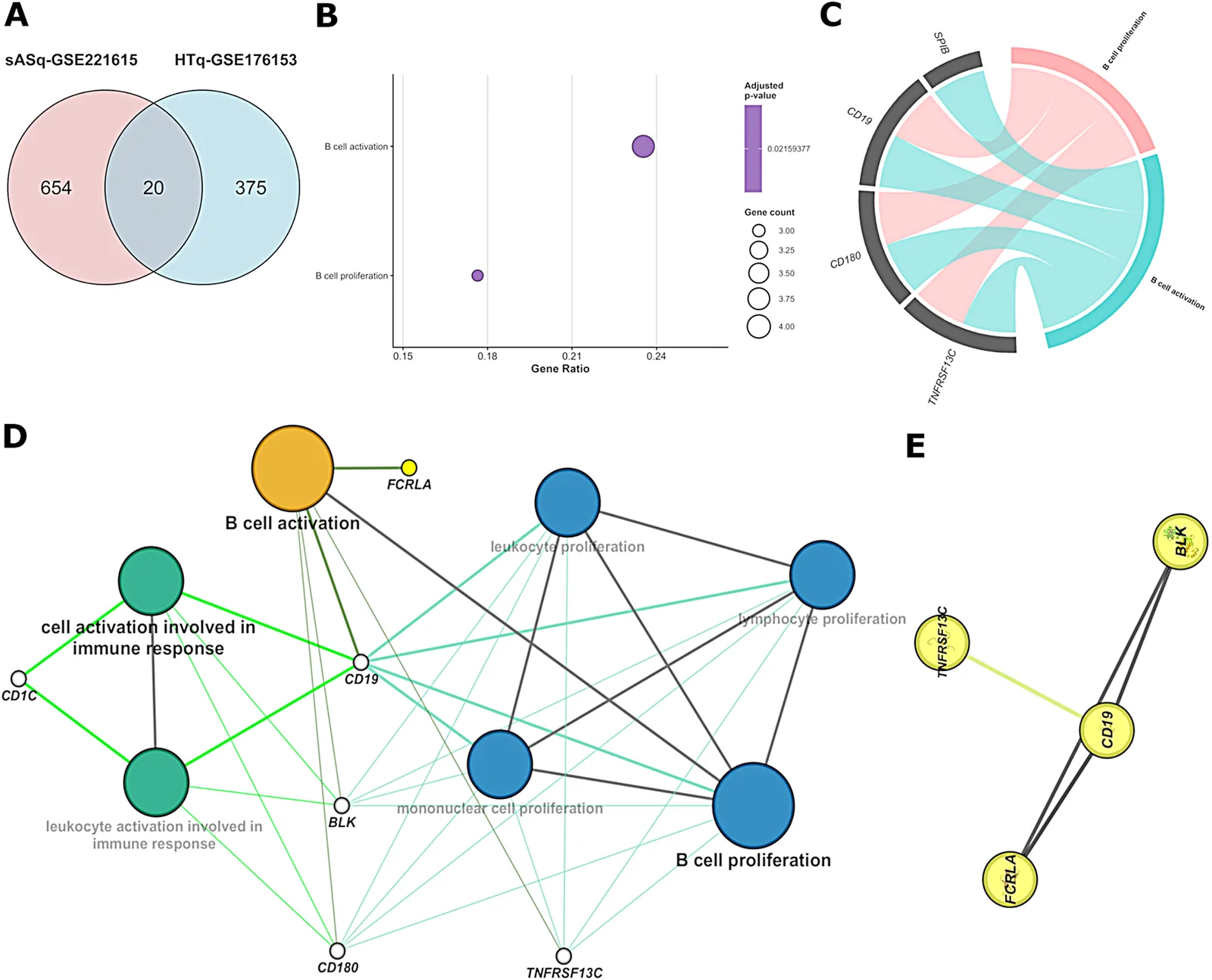
Identification and Functional Characterization of Shared Differentially Expressed Genes (SDEGs). **(A)** Venn diagram showing the refined intersection of DEGs within the female-specific cohorts (sASq- GSE221615 and HTq- GSE176153). **(B)** Dot plot representing Gene Ontology (GO) Biological Process (BP) enrichment analysis for the 20 SDEGs; dot size indicates gene count, while the color gradient reflects the adjusted significance level (p-adj <0.05). **(C)** Chord diagram illustrating the distribution of SDEGs across the top enriched GO BP terms; left sectors represent individual genes, and right sectors indicate biological processes; ribbon thickness denotes the gene’s contribution to a specific term. **(D)** Functional grouping and interconnection of GO BP terms visualized via the ClueGO plugin in Cytoscape (kappa score threshold: 0.6; minimum four genes per term). **(E)** Protein-protein interaction (PPI) network of female-restricted SDEGs generated using STRING through Cytoscape/STRINGApp. Nodes represent proteins and edges represent high-confidence protein–protein interactions with a combined STRING confidence score ≥0.85. Disconnected nodes were excluded for clarity.

### 3.2 Functional enrichment and network analysis of SDEGs

GO BP enrichment analysis of the 20 female-specific SDEGs revealed an enrichment of two immune-related pathways: B cell proliferation and B cell activation (p-adj <0.05; Figure 1B). This functional landscape was primarily represented by four genes, as illustrated in the chord diagram (Figure 1C): *TNFRSF13C* (TNF receptor superfamily member 13C), *CD180* (CD180 molecule), *SPIB* (Spi-B transcription factor), and *CD19* (CD19 molecule). Notably, a separate GO BP analysis of the upregulated SDEGs yielded consistent results (p-adj <0.005).

To further elucidate the biological interplay between the female-restricted SDEGs and the enriched GO BP terms, an interaction network was constructed using the ClueGO plugin in Cytoscape (Figure 1D). The network revealed an immune-related profile characterized by B cell activation, B cell proliferation, and cell activation involved in the immune response. Notably, *BLK*, *CD180*, and *CD19* were shared across all three biological processes. *TNFRSF13C* was associated with both B cell activation and proliferation, whereas *FCRLA* was linked to B cell activation.

Regarding the Cellular Component (CC) ontology, the SDEGs were significantly enriched in a single term: “external side of plasma membrane” (p-adj = 0.02).

To identify potential PPI among the female-restricted SDEGs, a network analysis was performed using the STRING database integrated via Cytoscape (Figure 1E). The resulting high-confidence PPI network comprised four genes: *CD19, BLK, FCRLA*, and *TNFRSF13C*. Based on node degree, *CD19* was the most highly connected node (degree = 3), while *BLK* and *FCRLA* showed intermediate connectivity (degree = 2).

Taken together, the integration of functional enrichment, ClueGO-based functional network analysis, and PPI topology highlighted six pivotal SDEGs, *TNFRSF13C, CD180, CD19, SPIB, BLK*, and *FCRLA*, associated with B cell proliferation and activation processes in females.

### 3.3 Identification of regulatory TFs

iRegulon analysis identified 7 TFs associated with the female-restricted upregulated SDEG set (Table 1). The highest-ranked regulons corresponded to *POU5F1* (POU class 5 homeobox 1), *SPI1* (Spi-1 proto-oncogene), and *MEF2A* (myocyte enhancer factor 2 A), with Normalized Enrichment Scores (NES) of 6.310, 6.227, and 5.933, respectively. Notably, *SPI1* showed associations with 10 SDEGs, including *BLK*, *TNFRSF13C*, and *CD19*, whereas *MEF2A* was associated with 8 SDEGs, including *BLK*, *FCRLA*, and *TNFRSF13C.* The predicted targets of SPI1 and MEF2A collectively included all six pivotal SDEGs (Figure 2A).

### 3.4 Discriminatory performance of pivotal SDEGs and TFs

The individual discriminatory performance of the six pivotal SDEGs was evaluated through ROC analysis (Supplementary Table 3). In the sASq-GSE221615 dataset, five of the six pivotal SDEGs showed AUC values ≥ 0.70 (Figure 2B), with *TNFRSF13C* displaying the highest AUC (0.835; 95% CI: 0.664–1.000), followed by *SPIB* (0.808; 95% CI: 0.626–0.990). In the HTq-GSE176153 cohort, all six pivotal SDEGs yielded an AUC of 1.00, reflecting complete separation of the three HT cases from the three controls in this dataset (Figure 2B). However, given the limited sample size, these estimates cannot be interpreted as evidence of diagnostic utility.

**FIGURE 2:**
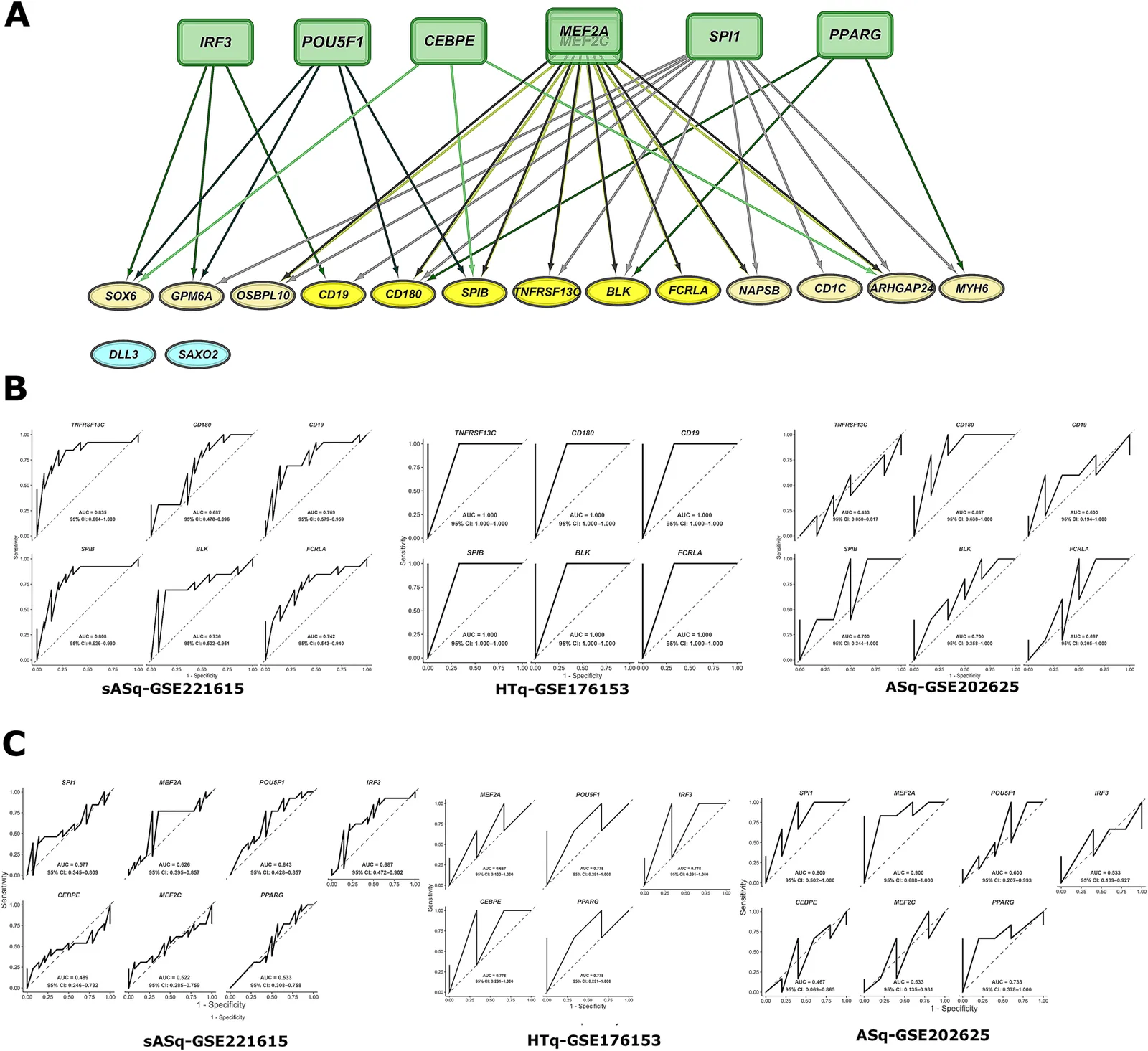
Regulatory Network Analysis and Classification Performance. **(A)** Transcriptional regulatory network of the female-restricted signature. Square nodes represent the predicted transcription factors (TFs), and circular nodes represent their targeted SDEGs. Yellow-highlighted nodes indicate the six pivotal SDEGs. Nodes highlighted in light blue indicate SDEGs that were not identified as targets for the predicted TFs. *MEF2A* and *MEF2C* have identical predicted target sets and are shown as overlapping square nodes with one shared set of edges for visualization purposes, avoiding redundant connections. **(B)** Receiver Operating Characteristic (ROC) curves evaluating the discriminatory performance of the six pivotal SDEGs (*TNFRSF13C, CD180, CD19, SPIB, BLK,* and *FCRLA*) across three datasets: (i) the sASq-GSE221615 cohort, (ii) the HTq-GSE176153 cohort, and (iii) the ASq-GSE202625 cohort of established coronary artery disease, used as an external proxy. **(C)** ROC curves showing the individual discriminatory performance of the seven predicted TFs across the same cohorts. AUC values and corresponding 95% confidence intervals are reported for each ROC analysis. *SPI1* and *MEF2C* were excluded from the HTq-GSE176153 analysis because their expression was undetectable across all samples in this dataset.

Due to the unavailability of a matching public cohort of sAS, the AS-GSE202625 dataset, comprising individuals with established coronary atherosclerotic disease, was used as an external proxy cohort. The female subset (ASq-GSE202625) included 11 samples (6 cases with coronary artery disease and five controls), with batch included as a covariate in the DESeq2 design. In this established disease context, *CD180* showed the highest individual discriminatory performance among the six pivotal SDEGs (AUC = 0.87; 95% CI: 0.64–1.00), followed by *SPIB* and *BLK* (both AUC = 0.70; Figure 2B).

Additionally, we evaluated the individual classification performance of the 7 TFs predicted by iRegulon (Figure 2C; Supplementary Table 3). In the sASq-GSE221615 cohort, all TFs showed limited classification performance, with AUC values below 0.70. In the HTq-GSE176153 cohort, *POU5F1*, *IRF3*, *CEBPE*, and *PPARG* showed AUC values of 0.78 (95% CI: 0.29–1.00), whereas *SPI1* and *MEF2C* could not be evaluated because their expression was undetectable across all samples. In the ASq-GSE202625 proxy cohort, *MEF2A, SPI1,* and *PPARG* showed AUC values of 0.90 (95% CI: 0.69–1.00), 0.80 (95% CI: 0.50–1.00), and 0.73 (95% CI: 0.38–1.00), respectively.

### 3.5 Immune infiltration analysis

The CIBERSORTx algorithm was employed to deconvolute the relative proportions of immune cell subpopulations in the female-specific datasets. All samples met the significance threshold (p < 0.05), ensuring that the inferred fractions were considered suitable for downstream analysis. While no significant differences in the relative abundance of immune cells were observed between the diseased and control groups (data not shown, Figures 3C,D), Spearman correlation analysis revealed associations between the expression of SDEGs and specific immune signatures (Figure 3A). In the HTq-GSE176153 dataset, the CD8^+^ T cell signature showed very strong positive correlations with *BLK* and *SPIB*. Conversely, the naïve CD4^+^ T cell signature was negatively correlated with *FCRLA*. In the sASq-GSE221615 dataset (Figure 3B), the memory B cell signature correlated negatively with *IFRD1*. Notably, moderate-to-strong positive correlations were identified between the activated NK cell, M2 macrophage, and resting mast cell signatures and several pivotal SDEGs, including *TNFRSF13C, BLK, SPIB, FCRLA*, and *CD19*. However, it is important to note that the estimated M2 macrophage fraction was notably smaller relative to the monocyte, T cell, and B cell populations (Figure 3C).

**FIGURE 3:**
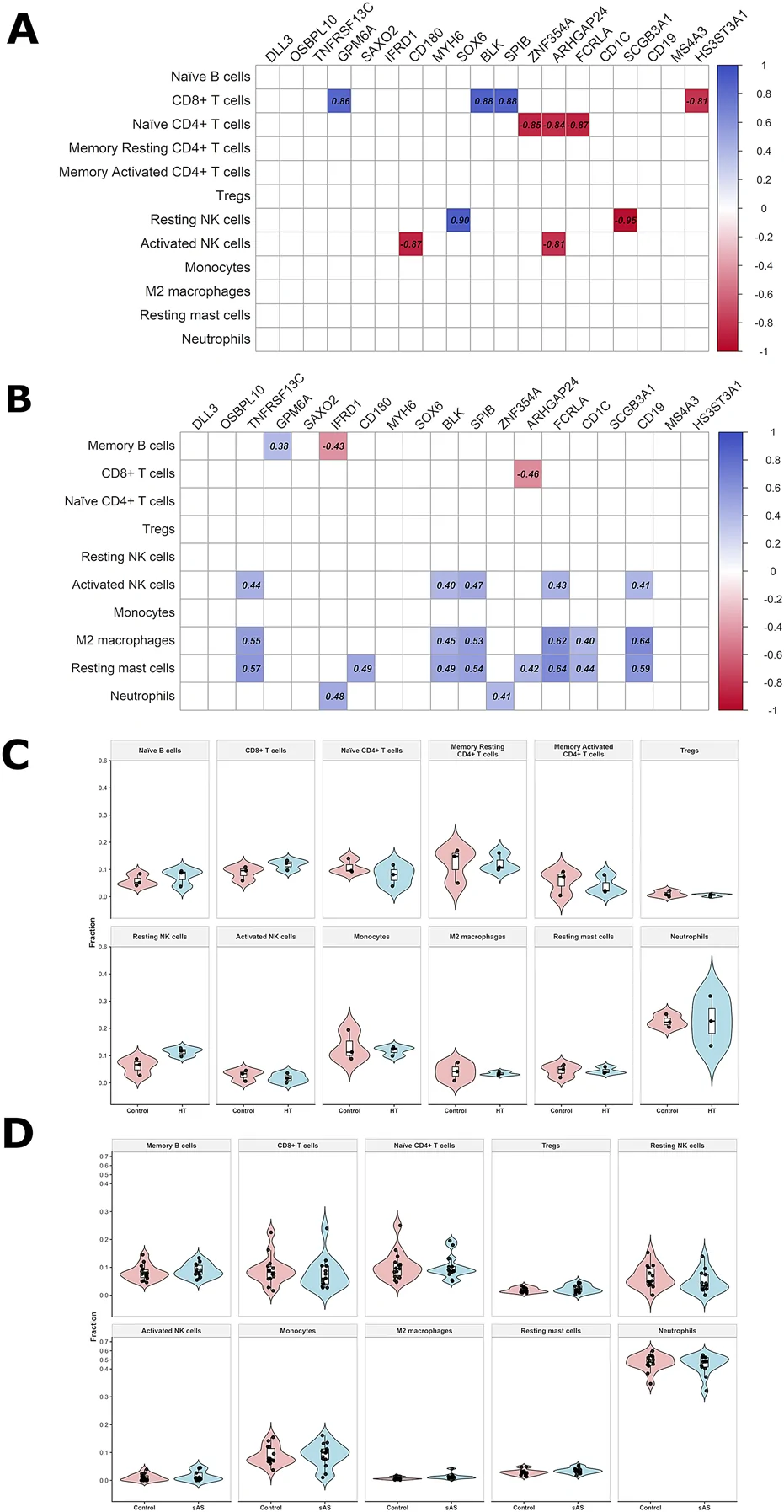
Immune Infiltration and Correlation Analysis. Assessment of the immune microenvironment and its association with SDEGs in the female-specific datasets. **(A,B)** Spearman’s rank correlation matrices between inferred immune cell fractions and SDEG expression in the **(A)** HTq-GSE176153 and **(B)** sASq-GSE221615 cohorts. Correlations were calculated using the Hmiscrcorr R package and visualized with corrplot. Positive correlations are indicated on a blue scale, while negative correlations are shown in red. Correlation coefficients are displayed only for statistically significant correlations (p < 0.05). Note: The NAPSB pseudogene was excluded from this analysis as it was not represented in the CIBERSORTx signature matrix. **(C,D)** Violin plots illustrating the relative abundance of major inferred immune populations between disease and control groups in the **(C)** HTq-GSE176153 and **(D)** sASq-GSE221615 cohorts.

### 3.6 TSHR gene expression profile in peripheral blood leukocytes

To contextualize the potential systemic effects of TSH signaling, we retrospectively evaluated TSHR expression across peripheral blood leukocyte populations. Our retrospective analysis confirmed that TSHR is expressed in peripheral blood T and B lymphocytes, as well as in monocytes (Supplementary Figure 2). Notably, across several independent datasets, *TSHR* expression was significantly higher in B cells (GSE136200, GSE51984, and GSE221091) or T cells (GSE51984, GSE60424, and GSE107011) when compared to the monocyte fraction. This expression pattern provides a rationale for further investigating direct TSH-mediated effects on adaptive immune cells in the context of HT and sAS.

### 3.7 TSHR signaling and transcriptomic profiling in murine macrophages

Although our systemic discovery pipeline identified a predominantly lymphocyte-centric signature, monocytes and macrophages are major cellular contributors to atherosclerotic plaque initiation and progression. To investigate the transcriptional consequences of TSHR signaling disruption in myeloid cells, we analyzed the GSE275079 dataset, comprising primary bone marrow-derived macrophages (BMDMs) isolated from myeloid-specific *Tshr* knockout mice (*Tshr*KO) and their respective wild-type (*Tshr* f/f) littermates following 24-h stimulation with 1 ng/mL TSH (Zhang et al., 2025). We performed functional enrichment analysis of the downregulated DEGs to assess whether TSH–TSHR signaling disruption is associated with immunological processes also observed in our human analysis. The top 30 enriched terms included leukocyte/mononuclear cell proliferation, regulation of inflammatory response, immune response-regulating/activating signaling pathway, chemotaxis and migration (Figure 4A), indicating that TSHR signaling disruption is associated with transcriptional changes in pathways involved in immune activation and inflammatory responses.

**FIGURE 4:**
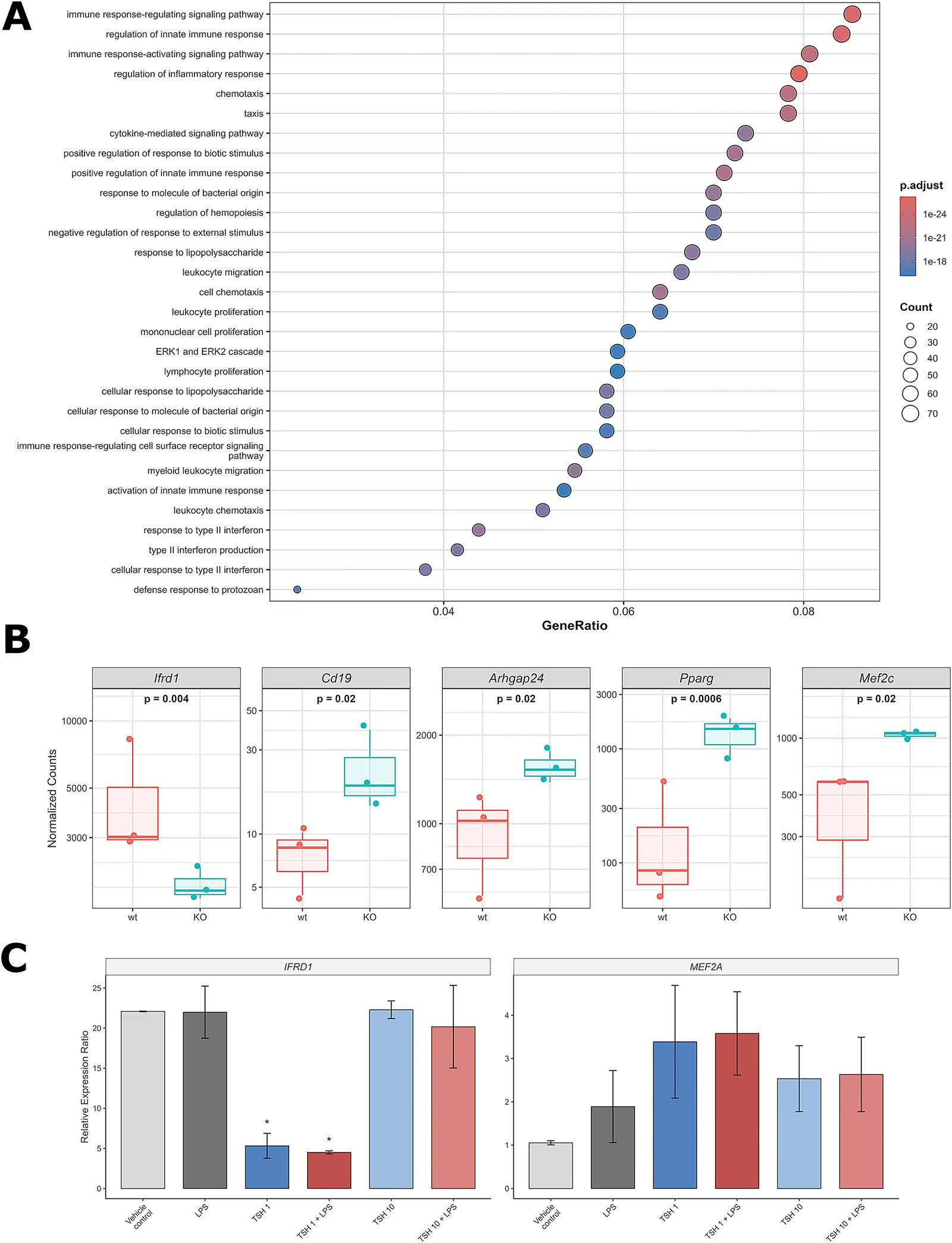
TSH signaling in murine and human macrophages. **(A)** Gene Ontology (GO) Biological Process enrichment analysis of downregulated differentially expressed genes (DEGs) in TSH-stimulated TshrKO vs. Tshr f/f murine macrophages. **(B)** Boxplots showing normalized counts for *Ifrd1, Cd19*, *Arhgap24*, *Pparg,* and *Mef2c* in murine bone marrow-derived macrophages (BMDMs). Comparisons were made between TshrKO (KO) and Tshr f/f (WT) genotypes. Individual data points represent biological replicates; boxes indicate the interquartile range (IQR), horizontal lines indicate the median, and whiskers extend to 1.5 × IQR. p-values displayed in each panel correspond to nominal p-values derived from the DESeq2 Wald test. **(C)** Human THP-1 monocytes were differentiated into M0 macrophages and subsequently stimulated with vehicle (control), LPS alone (positive control), or TSH at two different concentrations (1 and 10 mU/mL) in the presence or absence of LPS for 24 h. The mRNA expression levels of IFRD1 and MEF2A were analyzed by RT-qPCR and normalized to GAPDH expression. Data are presented as Mean ± SEM of expression ratios. * Significantly different from the vehicle control (p-adj <0.05). Data were collected from two or more independent experiments, typically with two replicate wells per condition, with replicate wells averaged before statistical analysis. Visualizations were generated using ggplot2.

Furthermore, we examined whether any of the previously identified female-restricted SDEGs and the predicted TFs were differentially expressed following myeloid-specific TSHR deficiency. Among the genes represented in the murine dataset, *Ifrd1* displayed significantly lower expression in *Tshr*KO-derived BMDMs (p = 0.004), whereas *Cd19* (p = 0.02), *Arhgap24* (p = 0.02), *Pparg* (p = 0.0006), and *Mef2c* (p = 0.02), showed significantly higher expression compared with wild-type controls (Figure 4B). Eight genes were not represented in the murine dataset and therefore could not be evaluated. Together, these findings suggest that TSHR deficiency is associated with altered expression of genes shared between sAS and HT, providing further evidence for a potential link between TSHR signaling and systemic inflammatory pathways.

### 3.8 *IFRD1* and *MEF2A* expression in human macrophages

To evaluate whether TSH exposure alters gene expression within the human myeloid lineage, we performed an exploratory *in vitro* analysis in PMA-differentiated THP-1 macrophages. *IFRD1* (*TIS7*) was selected as a candidate of interest based on its differential expression across multiple transcriptomic datasets. Specifically, *Ifrd1* expression was found to be downregulated in *Tshr*KO-derived BMDMs (GSE275079, Figure 4B). In human datasets, *IFRD1* was upregulated in the HTq-GSE176153 dataset, whereas its expression was significantly lower in the sASq-GSE221615 dataset and in the external proxy dataset ASq-GSE202625 (p = 0.0025). Human THP-1 monocytes were differentiated into macrophages by incubation with PMA. Although various PMA concentrations and incubation times were initially evaluated (data not shown), a protocol consisting of a 72-h incubation with 50 nM PMA followed by a 48-h recovery period in control medium was ultimately selected to obtain M0 macrophages. Under our experimental conditions, *IFRD1* mRNA levels were significantly decreased following exposure to 1 mU/mL TSH alone (p-adj = 0.001) and in combination with LPS (p-adj <0.001; Figure 4C). Interestingly, these effects were not observed at the higher TSH concentration (10 mU/mL). The basis for this concentration-dependent response remains unclear and warrants further investigation.

Parallel evaluations were conducted for *MEF2A*, which was identified as a candidate transcriptional regulator of the female-restricted SDEG network and has also been implicated in the regulation of pro-inflammatory responses in macrophages (Zhang et al., 2026). Furthermore, although MEF2A typically acts as a cytoprotective factor that promotes the proliferation, differentiation, and survival of vascular endothelial and smooth muscle cells, it can exhibit detrimental functions under specific pathological conditions (Liu et al., 2022). In our *in vitro* setup, *MEF2A* mRNA levels did not differ significantly between any treatment group and the vehicle control (Figure 4C).

### 3.9 Comparative analysis of shared and distinct molecular pathways with autoimmune disorders

Growing evidence suggests that RA and SLE involve chronic activation of immune and inflammatory pathways, which has been associated with an increased incidence of AS in these patients (Ryu et al., 2019). To determine whether the transcriptomic signature identified in our study is unique to the HT-sAS axis or is also observed in systemic autoimmune conditions, we analyzed the overlap of differentially expressed genes between the female-specific HTq-GSE176153 dataset and independent SLE and RA datasets (Supplementary Table 4). GO BP enrichment analysis of the shared SDEGs (Supplementary Figure 3) revealed predominantly immune-related processes, with recurring enrichment of antiviral, interferon-mediated, and antimicrobial responses across the systemic autoimmune datasets. These enrichment patterns differed from the B cell proliferation and activation signatures identified in our primary HT-sAS analysis. These findings suggest that HT shares some transcriptional features with systemic autoimmune conditions, but with distinct biological processes associated with the HT-sAS axis in females.

Likewise, we explored the shared molecular signatures between sAS and AIT by intersecting DEGs from sAS-GSE221615 and AIT-GSE269497. This intersection yielded 32 SDEGs, of which 18 showed consistent expression patterns (12 co-upregulated and six co-downregulated). GO BP enrichment analysis of the co-upregulated and discordantly regulated gene sets identified significant enrichment across multiple biological processes (Supplementary Figure 4). However, the enriched terms were heterogeneous and largely driven by a small number of genes, with substantial redundancy among related biological categories. Thus, despite the statistical enrichment, these results did not reveal a clear and biologically coherent pathway shared between sAS and AIT. Of note, a female-specific sub-analysis could not be performed for this comparison because biological sex metadata were not available in the AIT-GSE269497 dataset.

## 4 Discussion

Although inflammation is a recognized hallmark of sAS, the specific molecular mechanisms underlying this response in the context of HT remain poorly understood. In this study, we utilized whole blood as a surrogate tissue to capture systemic immune alterations underlying both conditions. The shared transcriptomic signature between HT and sAS was evaluated through a sex-specific analytical framework. By focusing exclusively on female datasets, we reduced biological heterogeneity associated with sex, identifying 20 SDEGs according to our predefined screening criteria. Functional enrichment analysis of these SDEGs revealed a predominant involvement of adaptive immune pathways, driven by terms related to B cell proliferation and activation.

While clinical HT is not typically characterized by overt leukocytosis, reporting on peripheral lymphocyte counts remains mixed. For instance, Fang et al. (2024) observed higher lymphocyte counts in hypothyroid patients in the clinical setting of obstructive sleep apnea (Fang et al., 2024). Conversely, in AIT patients with thyroid dysfunction - including overt and subclinical HT - peripheral lymphocyte counts were lower, likely reflecting lymphocyte recruitment and migration into the inflamed thyroid tissue (Xue and Xu, 2025). In our cohorts, cell-type proportions estimated using the CIBERSORTx algorithm revealed no significant differences in the relative abundance of major immune cell subsets, including B cells, between diseased and control groups (Figures 3C,D), although subtle differences in cell composition cannot be excluded. Overall, these observations are consistent with a recent large-scale genome-wide meta-analysis that identified multiple HT risk loci that pleiotropically regulate peripheral blood cell counts - including lymphocytes and neutrophils - and circulating inflammatory proteins (Rand et al., 2025). The enrichment of genes involved in differentiation, activation, and regulation of myeloid and lymphoid blood cells, and regulation of inflammatory responses within these HT-associated loci is consistent with the immune-related component of the shared transcriptomic signature identified here.

The potential translational relevance of our findings was explored through assessment of the discriminatory performance of the six pivotal SDEGs (*TNFRSF13C, CD180, CD19, SPIB, BLK,* and *FCRLA*). ROC analyses showed that several of these genes displayed discriminatory capacity in the discovery cohorts, although these findings do not constitute independent validation. Importantly, evaluation in the external ASq-GSE202625 cohort showed that part of this discriminatory pattern was retained in established atherosclerotic disease, with *CD180*, *SPIB*, and *BLK* showing measurable discriminatory capacity. These findings suggest that components of the identified transcriptomic regulatory axis may capture molecular features that persist beyond the subclinical stage, while their diagnostic or predictive utility requires confirmation in larger, disease-specific cohorts.

Our network analysis identified a high-confidence module of interconnected hub genes, *CD19, BLK, FCRLA,* and *TNFRSF13C,* highlighting the prominent involvement of B-cell-associated molecular pathways in the female HT-sAS axis. Notably, these genes encompass key components of B-cell signaling and maturation, further supporting the enrichment of adaptive immune processes observed in the functional analysis. The involvement of B cells in the progression of AS is complex and has been recently reviewed elsewhere (Sage et al., 2019; Ma et al., 2021; Pattarabanjird et al., 2021). To date, both proatherogenic and antiatherogenic properties have been assigned to B cells (Sage et al., 2019; Pattarabanjird et al., 2021). The importance of B cell responses in atherogenesis is highlighted by genome-wide association and transcriptomic data pinpointing the proliferation and activation status of B cells as important factors in the risk of CV disease (Huan et al., 2013). The analysis of whole blood gene expression profiles of coronary heart disease case/control individuals from the Framingham Heart Study revealed differential expression modules that were found to be enriched for B-cell immune genes. By integrating various data sources, they found two key driver genes, *SPIB* and *TNFRSF13C*, exhibiting strong effects on these modules (Huan et al., 2013).

B-cell activating factor (BAFF) critically regulates B-cell differentiation, proliferation, and survival by signaling through three distinct receptors: BAFF-R (encoded by *TNFRSF13C*), BCMA (*TNFRSF17*), and TACI (*TNFRSF13B*) (Ueland et al., 2026; Pattarabanjird et al., 2021). Beyond its classical roles, BAFF binding to BAFF-R has been shown to activate the NLRP3 inflammasome within B cells, triggering the secretion of pro-inflammatory IL-1b (Lim et al., 2020). While B-cell depleting therapies such as belimumab - a neutralizing monoclonal antibody that prevents BAFF from binding to BAFF - are well-established in systemic autoimmunity, their precise CV implications are still being elucidated (Sage et al., 2019; Ueland et al., 2026). Furthermore, BAFF signaling extends beyond the B-cell compartment to modulate T cells and monocytes, thereby influencing broader inflammatory pathogenesis (Luo et al., 2025).

BLK, a Src-family tyrosine kinase essential for B-cell receptor signaling (Cheng et al., 2019), is also expressed in a variety of cell types, including immune and non-immune cells (Li et al., 2024). BLK has been linked to susceptibility to autoimmune disorders (Li et al., 2024), SLE (Hong et al., 2026), and autoimmune thyroid diseases (Cheng et al., 2019). Notably, a recent study has identified BLK as a key pleiotropic gene linking Sjögren’s Disease to increased CV and stroke risk (Yi et al., 2025).

Fc receptor-like (FCRL) proteins play a role in promoting and regulating the immune response through their immunoreceptor tyrosine-based motifs. FCRL proteins are expressed mainly by B cells, suggesting a role in B-cell responses (Haroun et al., 2026). To our knowledge, no specific role for FCRLA has been described in the context of CV disease, making *FCRLA* a novel candidate within the HT-sAS axis.

Intriguingly, these B-cell hub genes displayed a significant positive correlation with the M2 macrophage signature in the sASq dataset. While M1 macrophages are traditionally associated with plaque progression, the M2 phenotype is linked to remodeling and repair, debris clearance, and AS regression (Bi et al., 2019). Although estimated M2 relative abundance was low, this exploratory association warrants further investigation given the reported functional interactions between B-cell signaling and macrophage polarization (Huang et al., 2025).

To explore the upstream regulatory mechanisms underlying the female-restricted signature, we performed an *in silico* TF prediction analysis using iRegulon. This analysis identified multiple candidate regulators, among which *SPI1* and *MEF2A* showed broad predicted regulatory coverage of the pivotal SDEG set, supporting their potential involvement in the transcriptional program shared by HT and sAS. The individual classification performance of the TFs was subsequently assessed by ROC analysis. Several TFs showed AUC values above 0.70 in the HTq-GSE176153 discovery cohort, although these estimates should be interpreted cautiously given the small sample size. While neither TF showed discriminatory capacity in the sASq-GSE221615 discovery cohort, *MEF2A, SPI1,* and *PPARG* showed discriminatory capacity in the external ASq-GSE202625 proxy cohort representing established coronary artery disease. Together, these exploratory findings highlight *SPI1*, *MEF2A*, and *PPARG* as candidate regulators of interest in the shared transcriptomic phenotype, while their clinical relevance remains to be established in larger, independent cohorts.

Accumulating evidence supports that chronic systemic inflammation, as characteristically observed in systemic autoimmune diseases such as SLE and RA, is directly linked to the accelerated development of AS (Ahlers et al., 2025). Crucially, traditional CV risk factors like age, dyslipidemia, and hypertension alone fail to fully account for this heightened risk (Sage et al., 2019). Several mechanisms have been implicated in mediating autoimmunity-associated CV risk, either directly or by modulating other risk factors in a complex interplay. Key drivers of accelerated AS in these patients include endothelial dysfunction, pro-inflammatory cytokine signaling, and aberrant leukocyte function (Porsch and Binder, 2024). To assess the clinical specificity of our findings, we contrasted the HT-sAS signature against systemic autoimmune disorders (SLE and RA). While these conditions showed recurrent enrichment of type I interferon, antiviral, and antimicrobial responses, these pathways were not prominent in the HT-sAS axis, which instead showed enrichment related to B cell proliferation and activation. This difference in pathway enrichment suggests that the HT-sAS transcriptional signature may reflect biological processes distinct from those characterizing systemic autoimmune disorders.

Both shared and distinct mechanisms may increase the risk of AS in patients with HT, either directly or through interactions with other CV risk factors. In women, age and reproductive hormonal status may modify CV risk (D’Costa et al., 2025), while age-related changes may also influence thyroid function (Hayashi et al., 2026). Especially in HT secondary to AIT, the chronic low-grade inflammatory state associated with thyroid autoimmunity, together with potential immune-mediated effects on the vasculature, could contribute to endothelial dysfunction and sAS. A recent bioinformatics study at the tissue level reported that genes jointly associated with AS and AIT are predominantly involved in antigen processing and presentation and revealed elevated levels of memory B cells within the affected tissues (atherosclerotic plaques and thyroid samples) (Ma et al., 2025). In contrast, our findings in peripheral blood showed significant enrichment across multiple biological processes but did not recapitulate the antigen processing and presentation signature reported at the tissue level. This difference may reflect the distinct biological compartments analyzed and raises the possibility that circulating metabolic or hormonal factors, including TSH, may contribute to the molecular overlap between HT and sAS beyond mechanisms specifically related to autoimmune thyroiditis.

Extra-thyroidal TSHR expression is widespread, spanning endothelial cells, vascular smooth muscle cells, and peripheral blood cells (Alomair et al., 2024). It has been demonstrated that TSH can promote macrophage vascular inflammation and AS by thyroid hormone-independent mechanisms (Yang et al., 2019). Recently, myeloid-specific *Tshr* deletion in mice demonstrated that TSH-TSHR signaling promotes macrophage M1 polarization and pro-inflammatory cytokine secretion (Zhang et al., 2025). Functional enrichment of downregulated DEGs in *Tshr*KO murine macrophages (GSE275079) revealed a disruption of inflammatory responses, cytokine-mediated signaling, and leukocyte proliferation. Notably, the enrichment of leukocyte and mononuclear cell proliferation-related processes showed a convergence with the immune-related proliferative signature observed in human HT–sAS cohorts. Although the candidate signature is predominantly associated with B-cell-related processes, the contribution of myeloid cells to atherosclerotic inflammation represents an additional biological context relevant to the HT-sAS relationship.

The transcriptional behavior of *IFRD1* across human cohorts, murine models, and *in vitro* assays underscores a context-dependent regulatory pattern rather than a uniform directional response. In primary BMDMs from the Tshr-knockout model stimulated with TSH (GSE275079), *Ifrd1* expression was significantly lower in TshrKO macrophages compared to wild-type controls, suggesting that TSHR signaling is involved in the regulation of *Ifrd1* expression in macrophages. This observation is consistent with the upregulation of *IFRD1* observed in the context of a systemic hypothyroid state (HTq-GSE176153). Conversely, the sASq and ASq datasets capture transcriptional profiles associated with chronic inflammatory and vascular disease states, in which *IFRD1* was downregulated. In differentiated human THP-1 macrophages, TSH exposure significantly reduced *IFRD1* mRNA expression, both in the absence and presence of LPS. Biologically, IFRD1 is responsive to inflammatory stimuli and has been implicated in the regulation of NF-κB/p65-dependent transcriptional activity and participates in tissue homeostasis across various cell types (Iezaki et al., 2016; Xie et al., 2018). More recently, *Ifrd1* has been identified as a master regulator of paligenosis, a conserved cellular program enabling cell cycle reentry and tissue adaptation following injury (Miao et al., 2020). To the best of our knowledge, this study provides an initial indication of TSH-associated regulation of *IFRD1* in human macrophages, highlighting the need for further functional investigations.

While our study provides novel insights into the shared transcriptomic architecture of HT and sAS in women, several limitations should be acknowledged. First, the limited sample sizes inherent to the publicly available datasets, particularly the HTq dataset, may reduce statistical power and limit the identification of SDEGs. Accordingly, the resulting transcriptomic signature should be regarded as exploratory and hypothesis-generating and requires further evaluation in larger, independent cohorts. Second, the original datasets were not explicitly designed to explore cross-disease mechanisms; consequently, critical clinical metadata, such as thyroid hormone concentrations in sAS patients and carotid intima-media thickness in HT patients, were unavailable. Similarly, individual-level information on BMI, glucose levels, and lipid parameters was not available for the sAS patients and therefore could not be incorporated as sample-level covariates in the differential expression models. Additionally, information on menopausal status and hormone replacement therapy was unavailable, limiting our ability to assess the potential contribution of reproductive and hormonal factors to the transcriptomic profiles. Furthermore, the lack of antibody profiling prevented us from fully disentangling the individual contributions of autoimmune inflammation versus hormonal dysregulation to the pathogenesis of HT. Although restricting our analysis to female-derived libraries minimized biological heterogeneity stemming from sexual dimorphism and aligned with the higher clinical prevalence of HT in women, this approach does not account for potential effects of menopausal status or hormone replacement therapy. Prospective studies with comprehensive endocrine, vascular, and metabolic characterization, including information on menopausal status and hormone replacement therapy, will be important to determine the clinical relevance and generalizability of the identified transcriptomic signature. Third, technical heterogeneity is an inherent limitation of cross-study integration; for instance, differences in HTS platforms across GEO datasets could introduce potential biases. Fourth, because transcriptomic profiling was performed on bulk whole blood, we cannot completely exclude the possibility that subtle, undetected shifts in circulating B-cell subpopulations contributed to the observed expression profiles. The resolution of bulk transcriptomic approaches is insufficient to exclude subtle changes in specific subpopulations or definitively distinguish cell-composition effects from cell-intrinsic transcriptional changes. Future studies using single-cell RNA sequencing or flow cytometry will be needed to further disentangle these possibilities. Finally, while the systemic signature was B-cell-dominated, we did not perform *in vitro* functional testing of the identified candidates in human lymphocytes. Given the established role of macrophages in atherosclerotic lesion progression, we explored TSH responsiveness within the myeloid lineage using GSE275079 and THP-1 macrophages. These experiments do not constitute functional validation of the B-cell-dominated transcriptomic signature, and because the myeloid models were male-derived, they cannot establish whether the observed responses are female-specific. Future studies incorporating functional validation in lymphocyte subpopulations, particularly B cells, as well as female-derived myeloid models, will be important to further evaluate the biological relevance and generalizability of these findings.

In conclusion, we identified a candidate gene signature in women shared between HT and sAS. The transcriptomic profile observed in hypothyroid women was characterized by enrichment of B-cell-associated processes, including proliferation and activation, distinct from the classic interferon-dominated cascades observed in systemic autoimmune disorders. This altered transcriptomic profile in peripheral whole blood may reflect altered immune-cell states and/or subtle changes in cellular composition associated with early atherogenesis. The identification of TFs associated with this shared signature further expands the molecular framework linking HT and sAS in women. This signature provides a basis for further investigation of CV risk in women with thyroid dysfunction and warrants validation in prospective longitudinal studies to assess its clinical and prognostic relevance.

## Data Availability

The datasets presented in this study can be found in online repositories. The names of the repository/repositories and accession number(s) can be found in the article/Supplementary Material.
