# Supplementary material for: A whole-blood transcriptomic analysis of shared molecular mechanisms between subclinical atherosclerosis and hypothyroidism in women
Source: Front Bioinform. 2026 Oct 21;6:1932220. doi: 10.3389/fbinf.2026.1932220 (PMC13731356; doi:10.3389/fbinf.2026.1932220)
Supplement: Supplementary file 1 [file DataSheet1.pdf]

Supplementary Material

Supplementary Figure 1. Analytical workflow.

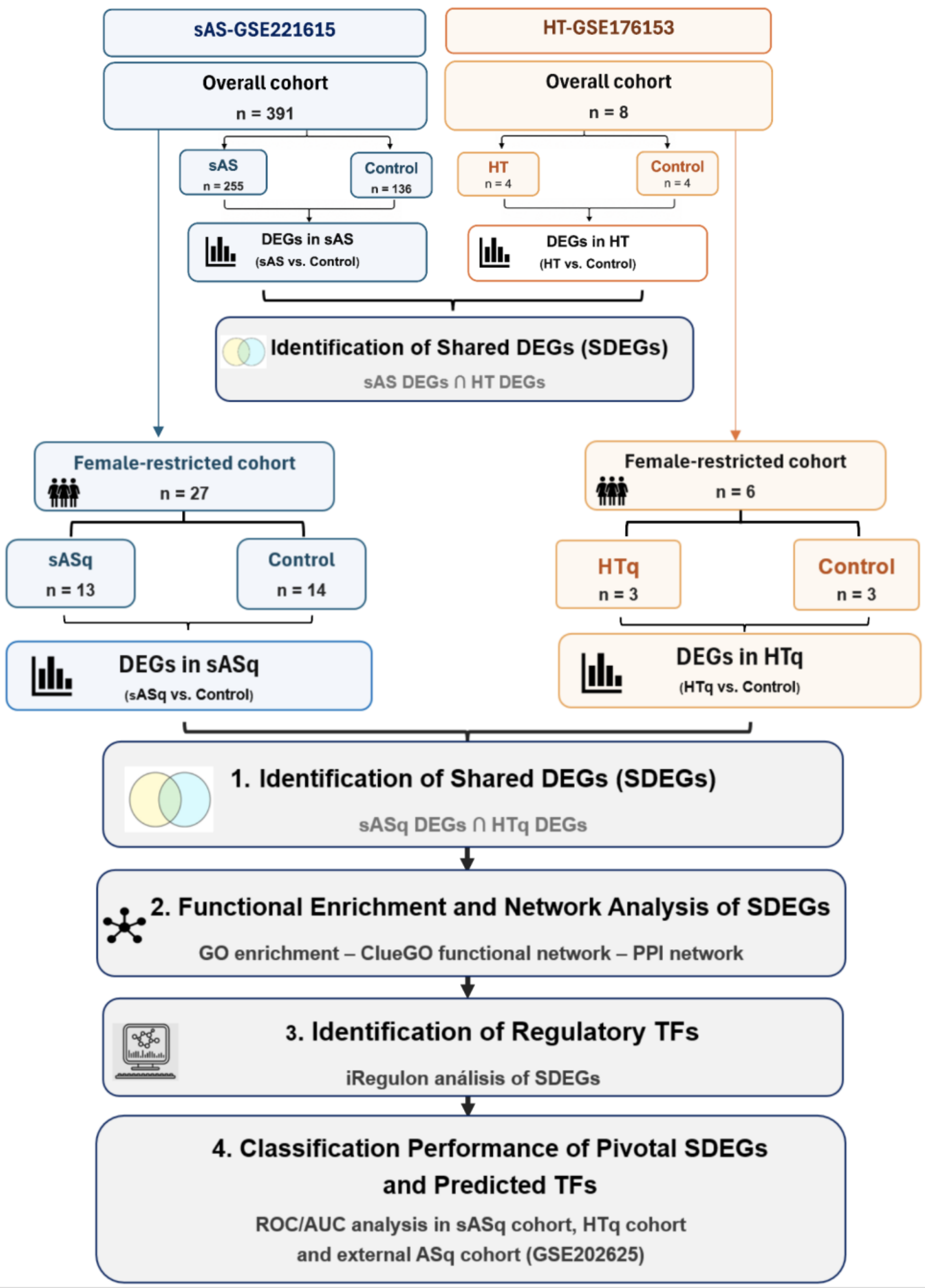

Schematic representation of dataset stratification and bioinformatic workflow. GSE221615 included 391 samples, and GSE176153 included 8 samples. Subsets restricted to female samples were used for the female-focused analyses (sASq, N = 27; HTq, N = 6). The workflow outlines: (1)

identification of SDEGs by dataset intersection; (2) functional enrichment (GO, ClueGO) and PPI network analysis; (3) regulatory TF prediction via iRegulon; and (4) exploratory classification performance evaluation using ROC/AUC analyses in the discovery cohorts and in an external proxy cohort (ASq-GSE202625). Abbreviations: sAS, subclinical atherosclerosis; HT, hypothyroidism; sASq, female subset of the sAS cohort; HTq, female subset of the HT cohort; DEGs, differentially expressed genes; SDEGs, shared differentially expressed genes; GO, Gene Ontology; PPI, protein-protein interaction; TFs, transcription factors; ROC/AUC, receiver operating characteristic/area under the curve.

Supplementary Table 1. Baseline characteristics of the study cohorts.

sAS cohort (GSE221615- sAS)

Characteristic	sAS (n=255)	Control (n=136)	p-value
Age, years, median (IQR)	51 (47–53)	47 (44–49)	<0.001
Female, n (%)	13 (5.1%)	14 (10.3%)	0.061

sAS female-restricted cohort (GSE221615- sASq)

Characteristic	sASq (n=13)	Control (n=14)	p-value
Age, years, median (IQR)	45 (44–49)	47.5 (44.25–49.5)	0.864

HT cohort (GSE176153-HT)

Characteristic	HT (n=4)	Control (n=4)	p-value
Age, years, median (IQR)	44.5 [36–51.5]	38 [36–40]	0.686
Female, n (%)	3 (75%)	3 (75%)	1.000

HT female-restricted cohort (GSE176153- HTq)

Characteristic	HTq (n=3)	Control (n=3)	p-value
Age, years, median (IQR)	51 [47.5–51]	40 [38–40]	0.400

Data are presented as Median [IQR]. p-values were calculated using the Wilcoxon rank-sum test. For the overall PESA Omics cohort (n=391), please refer to the original publication, where comprehensive baseline clinical characteristics are provided in the Supplementary Materials.

Supplementary Table 2. Sensitivity analysis.

Dataset	nominal DEGs (n)	FDR-significant DEGs (n)	FDR-significant DEGs
HT-GSE176153	921	5	*H3-3B; HLA-DRB1; DCLRE1C; SLC6A6; EIF3G*
sAS-GSE221615	355	8	*SEMA6B; GBP5; GPR15; IFI27; CXCL10; G0S2; AP3B2; OTOF*
HTq-GSE176153	375	16	*IFI44L; TLK2; TUSC2; DEFA4; COTL1; H3-3B; IFIT1; IFIT3; CYP4F3; PEX10; DCLRE1C; RASAL3; SLC6A6; EIF3G; PDLIM7; ISG15*
sASq-GSE221615	654	0	None

FDR-significant DEGs were defined using Benjamini–Hochberg false discovery rate-adjusted p-values (padj < 0.05), while retaining the |log2FC| > 0.26 effect-size criterion used in the primary analysis. Shared DEGs (SDEGs) were determined by intersecting the corresponding FDR-significant DEG lists. Under this stricter statistical threshold, no shared DEGs were identified in either the overall or female-specific intersection.

Supplementary Table 3. ROC AUC analysis of pivotal SDEGs and transcription factors.

Gene	HTq-GSE176153	sASq-GSE221615	ASq-GSE202625
TNFRSF13C	1.000 (1.000–1.000)	0.835 (0.664–1.000)	0.433 (0.050–0.817)
CD180	1.000 (1.000–1.000)	0.687 (0.478–0.896)	0.867 (0.638–1.000)
CD19	1.000 (1.000–1.000)	0.769 (0.579–0.959)	0.600 (0.194–1.000)
SPIB	1.000 (1.000–1.000)	0.808 (0.626–0.990)	0.700 (0.344–1.000)
BLK	1.000 (1.000–1.000)	0.736 (0.522–0.951)	0.700 (0.358–1.000)
FCRLA	1.000 (1.000–1.000)	0.742 (0.543–0.940)	0.667 (0.305–1.000)
POU5F1	0.778 (0.291–1.000)	0.643 (0.428–0.857)	0.600 (0.207–0.993)
SPI1	*	0.577 (0.345–0.809)	0.800 (0.502–1.000)
MEF2A	0.667 (0.133–1.000)	0.626 (0.395–0.857)	0.900 (0.688–1.000)
IRF3	0.778 (0.291–1.000)	0.687 (0.472–0.902)	0.533 (0.139–0.927)
CEBPE	0.778 (0.291–1.000)	0.489 (0.246–0.732)	0.467 (0.069–0.865)
MEF2C	*	0.522 (0.285–0.759)	0.533 (0.135–0.931)
PPARG	0.778 (0.291–1.000)	0.533 (0.308–0.758)	0.733 (0.378–1.000)

AUC, area under the receiver operating characteristic curve; CI, confidence interval; ROC analyses were performed individually for the six predefined pivotal SDEGs and the transcription factors predicted in the iRegulon analysis. The sASq-GSE221615 dataset included 27 samples (13 sAS and 14 controls), the HTq-GSE176153 dataset included 6 samples (3 HT and 3 controls), and the ASq-GSE202625 proxy dataset included 11 samples (6 CAD and 5 controls). Values are presented as AUC (95% CI). The 95% CIs were estimated using the DeLong method. * *SPI1* and *MEF2C* were not evaluated in the HTq-GSE176153 cohort because their expression was undetectable across all samples.

Supplementary Figure 2. Comparative analysis of *TSHR* gene expression across human peripheral blood cell subsets.

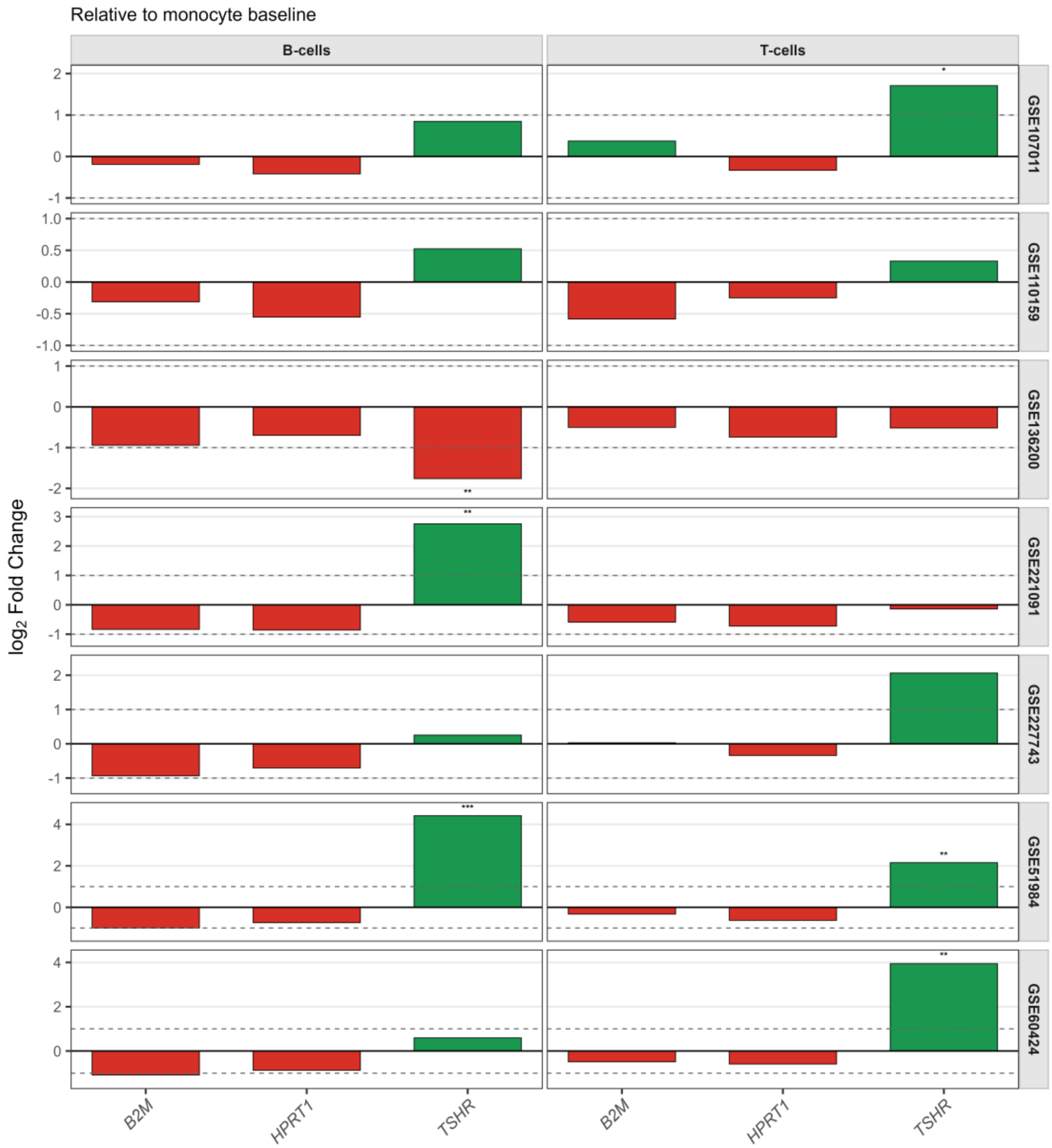

Bar plots illustrate the differential expression (log2 Fold Change) of *TSHR* in B and T lymphocytes relative to monocytes across seven independent datasets. The analysis was restricted to healthy control cohorts with a minimum of three biological replicates per group; sample sizes for B cells, T cells, and monocytes are indicated in parentheses for each dataset: GSE136200 (4, 8, 4), GSE110159 (4, 4, 4), GSE227743 (8, 18, 4), GSE51984 (5, 5, 5), GSE221091 (4, 9, 4), GSE60424 (4, 8, 4), and GSE107011 (16, 58, 12). The housekeeping genes *B2M* (beta-2-microglobulin) and *HPRT1* (hypoxanthine phosphoribosyltransferase 1) were included as negative controls for differential expression. Asterisks correspond to nominal significance values derived from the DESeq2 Wald test (* $p<0.05$, ** $p<0.01$, and *** $p<0.001$).

Supplementary Table 4. Comparison of SDEGs between female-specific HT and external autoimmune datasets.

			N SDEGs			N GO:BP terms		
Dataset	Subset	N patient/control *	All	Up	Down	All	Up	Down
GSE264125	SLE	10/10	72	9	5	58	na	na
GSE112087	SLE	58/54	165	44	15	90	5	0
GSE80183	SLE	12/4	61	18	3	42	46	na
GSE117769	RA	36/34	50	20	2	30	12	na
GSE90081	RA	12/12	97	26	10	38	0	0

Intersection of differentially expressed genes (DEGs) identified in the female-specific HT cohort (HTq-GSE176153) and external autoimmune datasets (SLE and RA), followed by overrepresentation analysis of Gene Ontology Biological Process (GO:BP) terms. * Analysis was strictly restricted to female samples across all cohorts. SLE: Systemic Lupus Erythematosus; RA: Rheumatoid Arthritis; All, Up, and Down indicate total, upregulated, and downregulated SDEGs, respectively, with concordant expression across both datasets; na: not applicable.

Supplementary Figure 3. Gene Ontology Biological Process enrichment analysis of shared genes between HT and systemic autoimmune datasets.

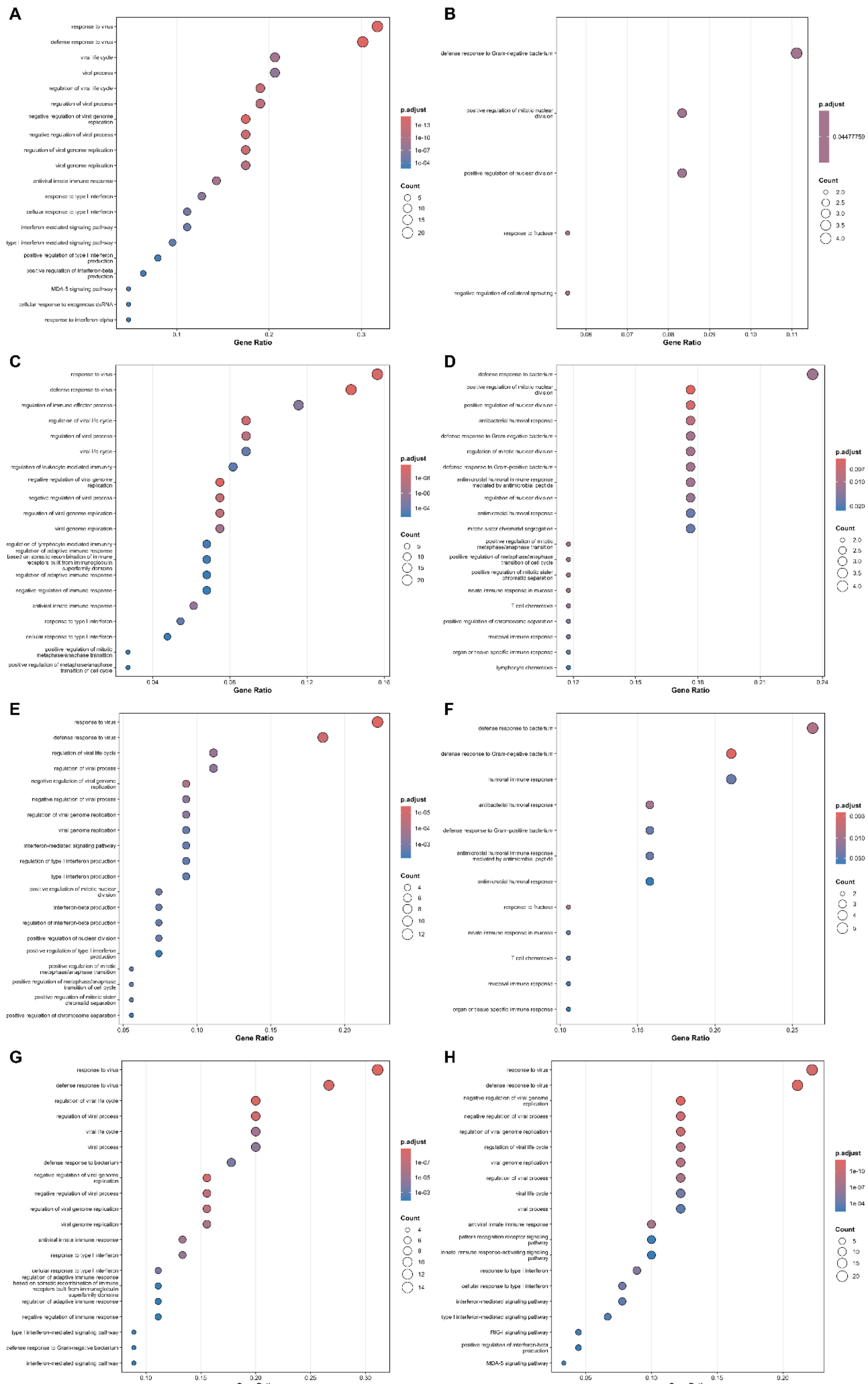

All comparisons were restricted to female samples across both the HT cohort (HTq-GSE176153) and the systemic autoimmune cohorts to minimize sex-related variability. The plots show the top 20 enriched Gene Ontology Biological Process (GO BP) terms identified using clusterProfiler::enrichGO from the shared differentially expressed genes (SDEGs) between HT and each systemic autoimmune dataset: (A) GSE264125, (B–C) GSE112087 (upregulated and total SDEGs, respectively), (D–E) GSE80183 (upregulated and total SDEGs, respectively), (F–G) GSE117769 (upregulated and total SDEGs, respectively), and (H) GSE90081.

Supplementary Figure 4. Gene Ontology Biological Process enrichment analysis of shared genes between sAS-GSE221615 and AIT-GSE269497.

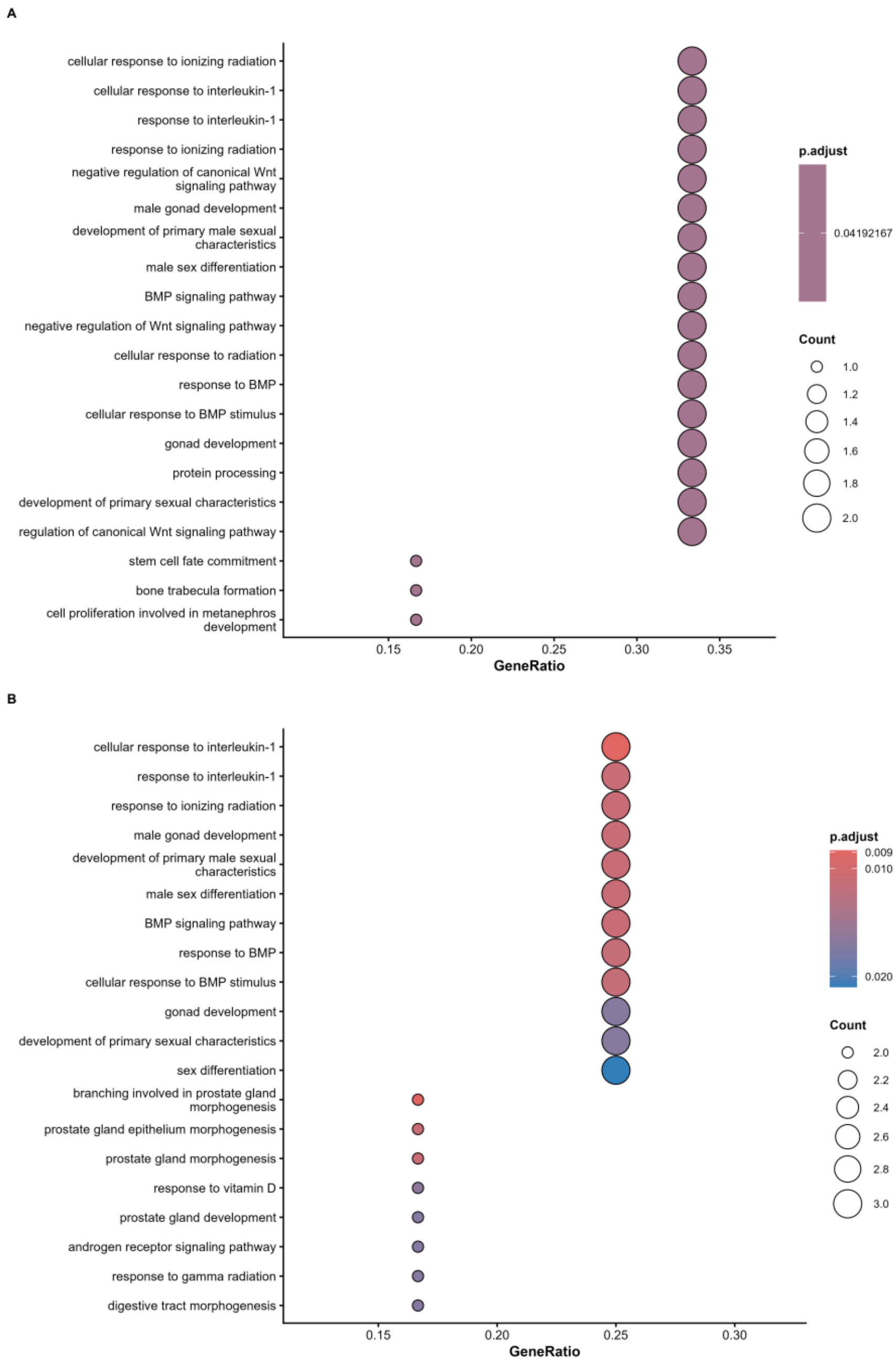

(A) Dot plot showing the top enriched Gene Ontology Biological Process (GO BP) terms among the upregulated SDEGs. (B) Dot plot showing the top enriched GO BP terms among all SDEGs. The x-axis represents the Gene Ratio, corresponding to the proportion of genes in each input gene set associated with the indicated GO term. Dot size indicates the number of genes contributing to each term, and dot color represents the adjusted *p*-value (Benjamini–Hochberg correction). Only the top 20 GO BP terms with adjusted $p < 0.05$ are shown.
